# Probabilistic modeling and risk characterization of the chronic aflatoxin M1 exposure of Hungarian consumers

**DOI:** 10.3389/fmicb.2022.1000688

**Published:** 2022-09-02

**Authors:** Zsuzsa Farkas, Kata Kerekes, Árpád Ambrus, Miklós Süth, Ferenc Peles, Tünde Pusztahelyi, István Pócsi, Attila Nagy, Péter Sipos, Gabriella Miklós, Anna Lőrincz, Szilveszter Csorba, Ákos Bernard Jóźwiak

**Affiliations:** ^1^Digital Food Institute, University of Veterinary Medicine Budapest, Budapest, Hungary; ^2^System Management and Supervision Directorate, National Food Chain Safety Office, Budapest, Hungary; ^3^Doctoral School of Nutrition and Food Sciences, University of Debrecen, Debrecen, Hungary; ^4^Institute of Food Science, Faculty of Agricultural and Food Sciences and Environmental Management, University of Debrecen, Debrecen, Hungary; ^5^Central Laboratory of Agricultural and Food Products, Faculty of Agricultural and Food Sciences and Environmental Management, University of Debrecen . Debrecen, Hungary; ^6^Department of Molecular Biotechnology and Microbiology, Institute of Biotechnology, Faculty of Science and Technology, University of Debrecen, Debrecen, Hungary; ^7^Food Chain Safety Laboratory Directorate, National Food Chain Safety Office, Budapest, Hungary; ^8^Institute of Nutrition, Faculty of Agricultural and Food Sciences and Environmental Management, University of Debrecen, Debrecen, Hungary; ^9^Analytical National Reference Laboratory, Food Chain Safety Laboratory Directorate, National Food Chain Safety Office, Székesfehérvár, Hungary; ^10^Analytical National Reference Laboratory, Food Chain Safety Laboratory Directorate, National Food Chain Safety Office, Budapest, Hungary

**Keywords:** AFM1, mycotoxin exposure assessment, long-term exposure, probabilistic method, consumer groups at risk

## Abstract

Aflatoxin contamination can appear in various points of the food chain. If animals are fed with contaminated feed, AFB1 is transformed—among others—to aflatoxin M1 (AFM1) metabolite. AFM1 is less toxic than AFB1, but it is still genotoxic and carcinogenic and it is present in raw and processed milk and all kinds of milk products. In this article, the chronic exposure estimation and risk characterization of Hungarian consumers are presented, based on the AFM1 contamination of milk and dairy products, and calculated with a probabilistic method, the two-dimensional Monte-Carlo model. The calculations were performed using the R plugin (mc2d package) integrated into the KNIME (Konstanz Information Miner) software. The simulations were performed using data from the 2018–2020 food consumption survey. The AFM1 analytical data were derived from the Hungarian monitoring survey and 1,985 milk samples were analyzed within the framework of the joint project of the University of Debrecen and the National Food Chain Safety Office of Hungary (NÉBIH). Limited AFM1 concentrations were available for processed dairy products; therefore, a database of AFM1 processing factors for sour milk products and various cheeses was produced based on the latest literature data, and consumer exposure was calculated with the milk equivalent of the consumed quantities of these products. For risk characterization, the calculation of hazard index (HI), Margin of Exposure, and the hepatocellular carcinoma incidence were used. The results indicate that the group of toddlers that consume a large amount of milk and milk products are exposed to a certain level of health risk. The mean estimated daily intake of toddlers is in the range of 0.008–0.221 ng kg^−1^ bw day^−1^; the 97.5th percentile exposure of toddlers is between 0.013 ng kg^−1^ bw day^−1^ and 0.379 ng kg^−1^ bw day^−1^, resulting in a HI above 1. According to our study, the exposure of older age groups does not pose an emergent health risk. Nevertheless, the presence of carcinogenic compounds should be kept to a minimum in the whole population.

## Introduction

Aflatoxins are secondary metabolites produced mainly by *Aspergillus flavus* (AFB1 and AFB2), *Aspergillus parasiticus* (AFB1, AFB2, AFG1, and AFG2) filamentous fungi. Aflatoxins are highly carcinogenic, genotoxic, teratogenic compounds that cause liver, kidney, and neurological damage and have immunosuppressive characteristics (IARC, 2012). Of the various types, the most common and most toxic is AFB1, which after digestion of contaminated feed, is metabolized in the liver of lactating animals to aflatoxin M1 (AFM1) and excreted in the milk and has the same adverse effects as AFB1 albeit in a lesser extent (Britzi et al., 2013; Marin et al., 2013). Aflatoxins are stable and resistant to heat and to most of the processing treatments, therefore they are present not only in milk but in all milk products. Moreover, as aflatoxins are bound to casein fractions, in some milk products such as cottage cheese and cheese, their concentration is even higher than in milk. Effective reduction of aflatoxin content of the end-product can only be done by prevention measures (Farkas et al., 2022).

Because of the genotoxic and carcinogenic nature of aflatoxins, the most important issue regarding their risk assessment is that no tolerable intake levels can be determined to which exposure levels could be compared. In such cases, Margin of Exposure (MoE) approach can be applied. MoE is the quotient of a reference value and the calculated exposure (Dybing et al., 2008). The reference value for aflatoxins is BMDL_10_ value derived from rat experiments for aflatoxin-induced liver cancer (lower confidence level of the smallest dose given in 95% probability that cause tumor in 10% of the animals). A more conservative risk characterization possibility is the application of a hazard index (HI), by using a safe dose proposed by Kuiper-Goodman (1990).

The European Food Safety Authority (EFSA) has evaluated the aflatoxin exposure of European consumers multiple times (EFSA, 2007, 2020) and concluded that the results are worrying for both AFB1 and AFM1, especially for younger consumers. The same results have been published in the studies of Kerekes et al. (2016) and Serraino et al. (2019), where AFM1 exposure of Italian consumers has been estimated. Health risk regarding AFM1 is also confirmed in other European studies, e.g., from Serbia by Djekic et al. (2020) and Milićević et al. (2021). Roila et al. (2021) also declare a public health concern related to the youngest consumers of the population of Central Italy regarding AFM1 exposure. As it has been proven in recent years, under favorable weather conditions for mycotoxin growth, aflatoxin contamination of grains can reach very high levels in Hungary as well (Ambrus et al., 2020; Szabó-Fodor et al., 2020). The European Commission recommends aflatoxins to be closely monitored in foodstuffs and the consumer exposure to be further studied. Aflatoxin exposure of Hungarian population has been estimated with a semi-deterministic method in the study of Kerekes et al. (2021). Deterministic assessments result in a characteristic score value for consumer exposure based on single values (average or high) of food consumption and contaminant concentration. For this method, worst-case scenarios must be assumed based on the precautionary principle. As a result, deterministic methods have a high uncertainty level and typically overestimate the risks. In case of the more refined probabilistic methods, the distribution of both consumption data and contamination concentrations is considered. The probability or frequency distributions characterize the range within which variables can occur and the probability that a variable meets a certain value. The results obtained also represent a distribution with a more realistic picture for consumer risk and with the elimination of likely overestimation of deterministic methods (Pieters et al., 2005).

As proper determination of aflatoxin consumer risk in European regions is of paramount importance, the aim of this study is to estimate the exposure level of the Hungarian population for AFM1 with a novel probabilistic method based on the available data. Risk characterization has also been conducted in order to identify the affected consumer groups regarding AFM1 exposure.

## Materials and methods

### Food consumption data

The Hungarian national food consumption data are deriving from the 2018–2020 survey of NÉBIH (Csizmadia et al., 2020a, b), which covered all regions of the country, four seasons, and all days of the week. The survey was part of EFSA’s Europe-wide EU MENU, or “What’s on the table in Europe?” Project and was conducted in accordance with the recommended, uniform methodology (EFSA, 2014). The participants were selected from the households included in the Central Statistical Office’s Household Budget and Living Conditions survey. During the program, two consumption days of 2,657 individuals between the ages of 1 and 74 were recorded with the help of dietitians by using a dietary software which was updated according to the methodology. Participants reported on food consumed the previous day in person or in the form of computer-assisted telephone interview. A picture book helped to estimate the amount of food consumed. The survey was supplemented by a questionnaire on body weight and height measurements, as well as food frequency and physical activity, covering a normal week in the 12 months prior to the interview. The recording of consumption habits for ages 1–9 was supported by a food diary.

Out of the 5,314 consumption days of the survey, a total of 5,145 milk consumption days (96.8%) were recorded, the frequency of sour cream and cream consumption was 54%, cheese consumption was recorded on 60.6% of the survey days, and kefir or yoghurt consumption was recorded on 24% of consumption days.

The food categories of the food consumption data were classified according to the FoodEx food classification system developed by EFSA (EFSA, 2015). The FoodEx classification system was created to link the data required for exposure estimation.

The consumption data of the Hungarian population were classified into 5 age categories (toddlers, children, adolescents, adults, and the elderly), following the EU MENU (EFSA, 2014) methodology. As the Hungarian EU MENU survey did not cover the age group of infants (0–1 years), this age group was not taken into account. The number of consumers is evenly distributed among the age groups.

### Aflatoxin M1 concentration data

The AFM1 data were obtained in the joint project “Estimation of the long- and short-term exposure of Hungarian consumers to aflatoxins present in dairy products and recommending risk management measures” (2018-1.2.1-NKP-2018-00002) of the University of Debrecen (DE) and NÉBIH carried out between 2018 and 2022. Out of a total of 2,608 AFM1 data measured in milk, the number of samples below the limit of detection (LOD) was 998 (38%), 1,103 results were between the LOD and the limit of quantification (LOQ) (42%), and 20% of the samples were above LOQ. NÉBIH collected the AFM1 concentration data in the framework of the Hungarian national monitoring program. While, the raw milk samples handled by the University of Debrecen (1985), originated from nine dairy farms participating in the project. The raw milk samples were analyzed by Aflatoxin M1 High Sensitivity ELISA (Romer Labs Inc., Tulln, Austria). The ones with a concentration above 20 ng kg^−1^ were subjected to a confirmatory HPLC analysis in the NÉBIH laboratory. For these samples, the results of the HPLC analysis were used for the calculations. NÉBIH contributed to the project by the data (623) of a nationwide monitoring survey focusing mainly on raw milk from small producers.

**Figure 1:**
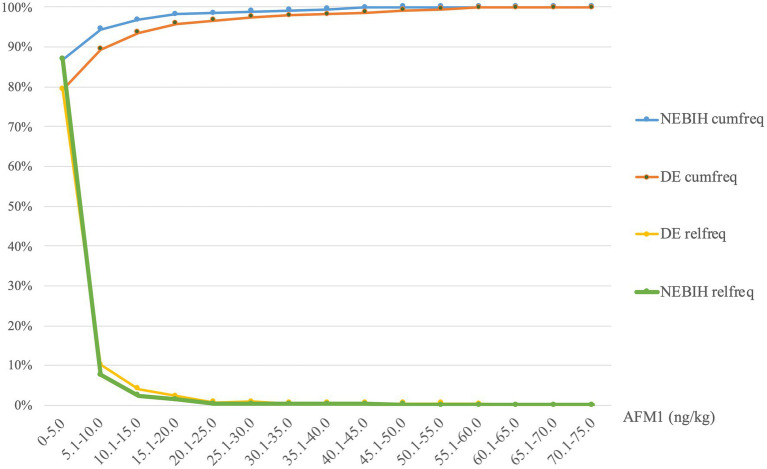
Relative and cumulative frequency distribution of DE and NEBIH AFM1 data.

Comparing the descriptive statistics (Table 1) and the relative frequency distributions (Figure 1) of the analytical results of two sources, the measured AFM1 contamination was found to be similar in the two datasets, which justifies the joint evaluation of the data.

**Table 1:** Descriptive statistics of the two data sources.

	DE project data	NEBIH project data
Count	1985	623
<LOQ	1,573	541
Minimum	2.90	5.00
Median	2.99	6.04
Mean	5.72	5.00
SD	7.75	4.34
95th percentile	18.64	11.00
Maximum	70.99	47.00

For a longer-term comparison, the relevant additional AFM1 data (1,115) were used from the 2011–2020 national monitoring survey of NÉBIH. Most of the measurements were performed by ELISA and HPLC methods on samples taken from milk from dairy farms, private producers, and a small proportion of mixed milk available in shops. Analysis of mycotoxin data was preceded by data cleaning steps. Laboratory staff were consulted in case of the dubious measurement results.

A limiting factor in the risk assessment of aflatoxins was the lack of data on processed dairy products. Following a recommendation from EFSA (EFSA, 2010), food categories should be excluded from the risk assessment for which the number of positive samples does not exceed 25 or for which the proportion of left censored data (<LOD/LOQ) is greater than 80%. The project provided adequate amount of milk AFM1 concentration data, but from the AFM1 monitoring results, only the milk samples met the above criterion, while the number of tests for processed dairy products was too small. Therefore, for processed dairy products, actual analytical results could not be taken into account for exposure estimation. Instead, values derived from AFM1 concentration data measured in milk were calculated, using the median of processing factors of dairy products found in the literature for each food category [e.g., yoghurt: 0.57 (Ibrahim et al., 2016; Barukcic et al., 2018), kefir 0.81 (Kamyar and Movassaghghazani, 2017), sour cream: 0.61 (Hashemi and Gholamhosseinpour, 2019) hard cheese: 5.6, (Manetta et al., 2009; Cavallarin et al., 2014; Pietri et al., 2016), semi-hard cheese: 4.6 (Oruc et al., 2007; Sakuma et al., 2016; Pecorelli et al., 2018, 2019), soft cheese: 2.3 (Govaris et al., 2001; Oruc et al., 2006; Cattaneo et al., 2013; Ibrahim et al., 2016), and fresh cheese: 2.2 (Cavallarin et al., 2014; Cetin et al., 2019)].

### Chronic exposure assessment

#### Intake assessment

Chronic exposure of the population for AFM1 was assessed by calculating the estimated daily intake (EDI), expressed as ng kg^−1^ bw day^−1^ with a probabilistic method. For AFM1 concentration, the distribution that best fit the occurrence data was used. For the consumption data, the observed individual mean (OIM) of the two consumption days of the individuals was used, which is recommended for long-term estimations.

First, all milk and dairy product consumption data were converted to milk equivalent using the processing factors specific to the given food category (Equations 1 and 2).

Intake of *e_1_*, …, *e_j_* foods expressed in g kg^−1^ bw *(B)* expressed in milk equivalent on a given *(n)* consumption day:


(1) $$B_{n}=\frac{\sum_{e=1}^{j}\left(m_{e}\times F_{e}\right)\;}{bw_{n}}$$

where *m_e_ =* mass (g) of the consumed *e* food on *n_i_* consumption day, *F* is the processing factor of *e* food, *bw* is the body weight of the person belonging to the given day of consumption, and


(2) $$F_{e}=\frac{C_{\mathrm{AFM}1_{e}}}{C_{\mathrm{AFM}1_{\mathrm{milk}}}}$$

where $C_{\mathrm{AFM}1_{\mathrm{milk}}}\;$is the concentration of AFM1 in the milk used to prepare the *e* food, $C_{\mathrm{AFM}1_{e}}$ is the value calculated from the median results in processed foods obtained in different experiments.

Then, the obtained total intake values per consumption day were expressed in kg^−1^ kg bw. The intake values of the consumers belonging to the two consumption days were averaged (OIM). The distribution of exposure values was calculated by multiplying the mean consumption amounts by the values taken from the distribution of the AFM1 concentrations (ng kg^−1^) with a probabilistic method.

#### Probabilistic method

For the probabilistic estimations, the GAMLSS and GAMLSS.dist packages (Rigby and Stasinopoulos, 2005) of the R statistical software were applied, using the maximum likelihood estimation. Different distributions were fitted to the analytical results above the LOD, which was weighted by the proportion of values below the LOD. Then, the distribution that gave the optimal fit was selected by the parameters describing the goodness of fit (AIC - Akaike’s Information Criterion, BIC - Bayesian Information Criterion, and Global Deviance). For AIC, BIC, and Global Deviance as well, the distribution with the smallest value should be considered as the best fit.

The goodness of the fit of the distributions was also evaluated by visual comparison of the histograms made from the data and the obtained distribution, as well as by examining the normality of the differences and using Q–Q plots. Both the residual statistics and the Q-Q plot examine the differences between the original and the fitted data, then compare the data set of the residuals to a standard normal distribution. Both methods use a correlation coefficient to compare data point by data point, how much they deviate from the normal distribution.

The best-fitting distribution was the four-parameter Box-Cox t (BCT), which is suitable for modeling aflatoxins-like positively or negatively skewed, slowly decaying data, with continuous distribution (Ferrari and Fumes, 2017; Rigby et al., 2019).

The selected distribution was fitted to the entire AFM1 data set. After that, the work has been continued with a probabilistic method, the two-dimensional Monte Carlo model (mc2d package in R) (Pouillot et al., 2015). The 2D Monte Carlo simulation repeatedly generates samples from the probability distribution fitted to the data by random sampling. This method provides a distribution of the expected exposure. The advantage of the Monte Carlo method is that the full spectrum of values below the distribution curve is used for the calculations. Values at both edges of the distribution play a particularly important role, which may play a key role in modeling.

The Monte Carlo model works with an external and an internal simulation loop. In the inner loop, the model performs the exposure calculation several times, randomly sampling consumption (OIM) and concentration data, calculating different percentiles of exposure from each iteration (this is the variability of exposure). The sum of these exposure calculations constitutes an iteration of the outer loop and results in an estimate of the distribution of exposures. The outer loop also runs several times, and since repeated iterations will necessarily result in different percentile values due to random sampling, their distribution is characterized by uncertainty in the estimation (EFSA, 2012).

In summary, the inner loop simulates the expected variability in daily exposures and the outer loop simulates the estimation uncertainty. At the end of the calculation series, the model characterizes the expected exposure of the population using the cumulative frequency distribution graph (Figures 3–6) as well as percentile values.

**Figure 2:**
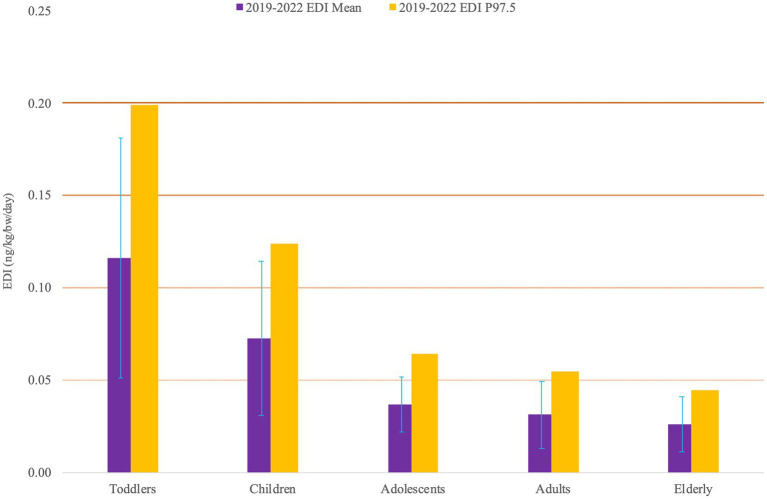
Median values of the mean and 97.5th percentile distributions.

**Figure 3:**
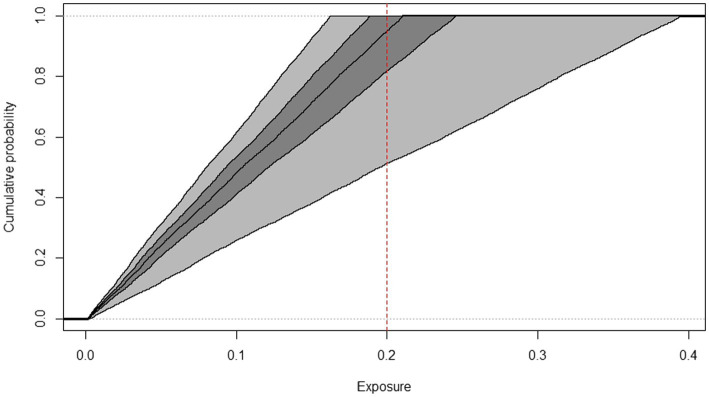
Cumulative frequency distributions of AFM1 exposure of toddlers, estimated by the 2D Monte Carlo method based on Box-Cox t distribution indicating the threshold used for HI calculation (dashed line), years 2019–2022.

**Figure 4:**
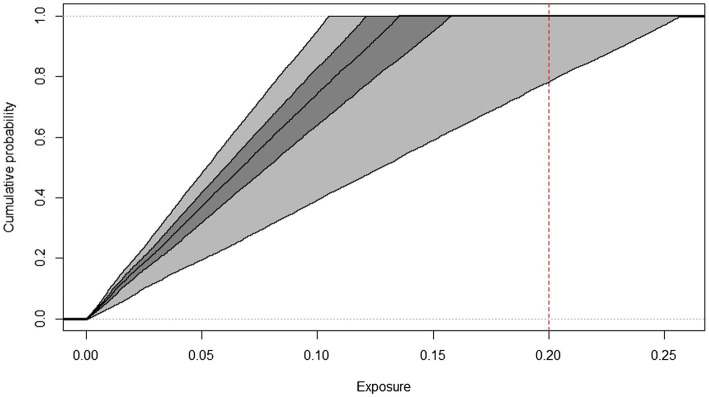
Cumulative frequency distributions of AFM1 exposure of children, estimated by the 2D Monte Carlo method based on Box-Cox t distribution indicating the threshold used for HI calculation (dashed line), years 2019–2022.

**Figure 5:**
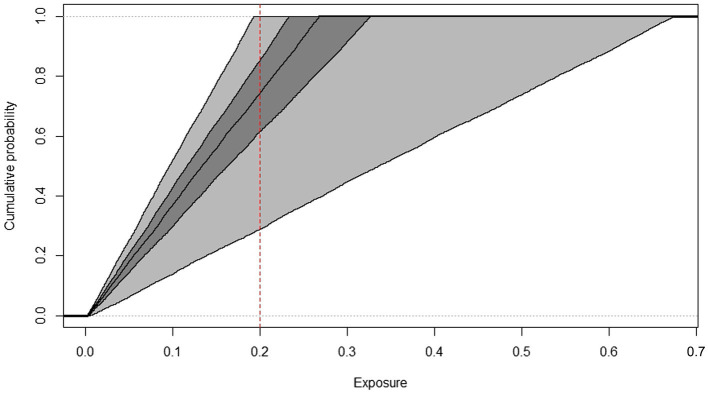
Cumulative frequency distributions of AFM1 exposure of toddlers, indicating the threshold used for HI calculation (dashed line), years 2011–2022.

**Figure 6:**
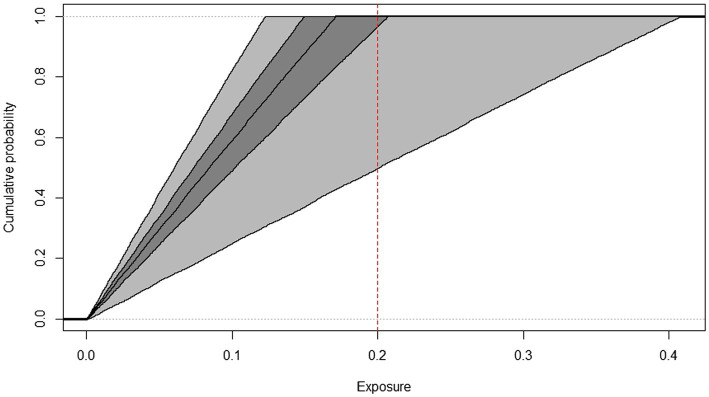
Cumulative frequency distributions of AFM1 exposure of children, indicating the threshold used for HI calculation (dashed line), years 2011–2022.

### Risk characterization

Based on the obtained exposure values, the MoE approach (Equation 3), the HI (Equation 4), and calculation of the incidence of hepatocellular carcinoma cases associated with aflatoxin exposure (Equation 5) were used to assess the risk of the Hungarian population. For the MoE method, the BMDL_10_ value of 0.4 μg kg^−1^ day^−1^ for AFB1 was taken into account by a multiplication factor of ten (4 μg kg^−1^ day^−1^) because AFM1 is a ten times less potent carcinogen than AFB1.


(3) $$\mathrm{MoE}=\frac{{\mathrm{BMDL}}_{10}}{\mathrm{EDI}}$$

To calculate the HI, the safe dose recommended by Kuiper–Goodman (0.2 μg kg^−1^ day^−1^) was used, which is the quotient of the tumor-causing dose in 50% of the animals and a safety factor of 50,000. Calculation of the aflatoxin HI for AFM1:


(4) $$\mathrm{HI}=\frac{\mathrm{EDI}\;\left(\mathrm{ng}\;{\mathrm{kgbw}}^{-1}{\mathrm{day}}^{-1}\right)}{0.2\;\mathrm{ng}\;{\mathrm{kgbw}}^{-1}{\mathrm{day}}^{-1}}$$

Exposure to aflatoxin increases the risk of developing HCC in the presence of chronic hepatitis B. The incidence of hepatocellular carcinoma associated with aflatoxin exposure was estimated, assuming hepatitis B prevalence of 0.7% in the Hungarian population (Horváth et al., 2018):


(5) $$R_{\mathrm{Hu}}=\left[\left(P_{\mathrm{HBV}+}\times \mathrm{HBV}+\right)+\left(P_{\mathrm{HBV}-}\times \left(1-\mathrm{HBV}+\right)\right)\right]\mathrm{xEDI},$$

where *R*_Hu_ is the risk of liver cancer incidence in the Hungarian population, HBV+ is the prevalence of chronic hepatitis B in the Hungarian population (0.007), *P*_HBV+_ is the probability of developing liver cancer in this part of the population (0.027), and *P*_HBV−_ is the probability of developing liver cancer in the rest of the population (0.002).

### Software used

The calculations were performed using KNIME (Konstanz Information Miner), a free, open-source data analysis software. KNIME has R integrations; therefore, R codes can be run within KNIME to perform computational tasks for which there is no built-in KNIME module. The developed exposure estimation methodologies can be modified, optimized, easily adapted to other contaminant-matrix combinations, or expanded with additional modules or data sources. To perform the calculation steps of the presented results, an integrated risk assessment KNIME workflow was created that is suitable for the processing of consumption data, deterministic and probabilistic exposure estimates, as well as for the characterization of exposure based on the obtained results.

## Results

### Exposure of the different consumer age groups (EDI)

The calculations were made based on data from the 2018–2020 food consumption survey, using the probabilistic method (2D Monte Carlo simulation), taking into account the median of processing factors and the Box-Cox t (BCT) distribution for AFM1 data. The exposure of different consumer age groups was compared, based on the median of the mean distribution and 97.5th percentile distribution of EDI values. The results are shown in Figure 2.

Taking into account the 95% range of estimation uncertainty, the mean exposure of toddlers is in the range of 0.008–0.221 ng kg^−1^ bw day^−1^ and it can be characterized by a median value of 0.116 ng kg^−1^ bw day^−1^ (standard deviation 0.065 ng kg^−1^ bw day^−1^) and a median value of 0.107 ng kg^−1^ bw day^−1^ of the median range. The 97.5th percentile exposure of toddlers is in the range of 0.013–0.379 ng kg^−1^ bw day^−1^, the median value is 0.199 ng kg^−1^ bw day^−1^.

The mean exposure of children ranges from 0.046 to 0.141 ng kg^−1^ bw day^−1^, with a median value of 0.073 ng kg^−1^bw day^−1^ (standard deviation 0.042 ng kg^−1^ bw day^−1^) and a median value of 0.067 ng kg^−1^ day^−1^. The 97.5th percentile exposure of children is in the range of 0.008–0.241 ng kg^−1^ bw day^−1^, the median value is 0.124 ng kg^−1^ bw day^−1^.

The distribution of the mean exposure of adolescents falls between 0.022 and 0.071 ng kg^−1^ bw day^−1^ (SD 0.021 ng kg^−1^bw day^−1^) with a median of 0.037 ng kg^−1^ bw day^−1^. The 97.5th percentile range is between 0.038 and 0.125 ng kg^−1^ bw day^−1^, the median of the 97.5th distribution is 0.064 ng kg^−1^ bw day^−1^.

Adults and the elderly have a slightly lower exposure level than adolescents. The medians of the mean exposures are 0.031 and 0.026 ng kg^−1^ bw day^−1^; the medians of the 97.5th percentile exposures are 0.055 and 0.045 ng kg^−1^ bw day^−1^, respectively.

Thus, the highest exposure values can be observed at the youngest and the lowest exposure values at the oldest age groups. However, the relationship is not direct between age and intake, but between the average body weight observed in different age groups (typically increasing by age) and intake, as exposure values are given per kilogram of body weight.

### Risk characterization of AFM1 intake [MoE, HI, hepatocellular carcinoma incidence (HCCi)]

Three evaluation methods were used for risk characterization. All methods are accepted in international practice, although the application of the HI and the MoE approach somewhat contradicts the fact that no safe tolerable daily intake can be established for genotoxic and carcinogenic compounds. Yet both methods are based on a limit value compared to which some aflatoxin intakes are considered riskier and others less risky. In any case, as the EDI alone does not provide sufficient information to judge whether the level of exposure can be considered low or high, these methods help to assess the level of risk.

Dividing the result of the exposure estimates (EDI) by a “safe dose” gives a dimensionless ratio. The extent of the risks is proportional to the results obtained and is considered to be of concern at values of 1 or higher. HI values calculated from the median of mean distribution of daily intake values indicate that the risk from exposure is not considered to be of concern in neither of the age groups (Table 2). However, in the case of toddlers, at the 97.5th percentile value (large consumers), the exposure is reaching the level considered as not safe.

**Table 2:** Mean and 97.5th percentile (P97.5) hazard index (HI) values of the different age groups for 2019–2022, calculated with a probabilistic method.

Age groups	HI mean	HI P97.5
Toddlers	0.58	1.00
Children	0.36	0.62
Adolescents	0.18	0.32
Adults	0.16	0.27
Elderly	0.13	0.22

The cumulative frequency distributions (median and confidence intervals) of AFM1 exposure of toddlers and children from 2019 to 2022 AFM1 concentration data calculated with the two-dimensional Monte Carlo simulation are shown in Figures 3, 4. In order to have a better understanding of the proportion of consumers at risk in these two age groups, the threshold used for HI calculation is indicated on the figures. The middle line represents the median exposure distribution, the dark-gray area is the 50%, the light-gray area is the 95% uncertainty interval.

Another option for characterizing the risk from AFM1 intake is the MoE. For aflatoxins, the BMDL_10_ value derived from AFB1-induced liver cancer studies in rats (400 ng kg^−1^ day^−1^) may be used as a reference value, which can be used for AFM1 converted by a factor of ten. Results below 10,000 are of concern, MoEs of 10,000 or greater are indicating low or no risk to public health.

The average and 97.5th percentile MoE estimates of the intake values calculated by probabilistic method from the 2019–2022 concentration and 2018–2020 food consumption data were compared by age groups (Table 3).

**Table 3:** Mean and 97.5th percentile MoE values of the different age groups for 2019–2022, calculated with a probabilistic method.

Age groups	MoE mean	MoE P97.5
Toddlers	34,483	20,101
Children	55,096	32,258
Adolescents	108,401	62,208
Adults	127,389	72,860
Elderly	152,672	89,087

The results from the MoE assessment provide us with a less worrying picture than the output values of the HI calculation. The limit of considerable risk (10,000) was not reached by any of the age groups. Therefore, no significant risk can be identified with this risk characterization methodology.

A third method used for risk characterization is the estimation of the contribution of mean and high AFM1 intake in a given population to the incidence of HCCi, i.e., the incidence of new cases in a given population over a given period of time (Table 4).

**Table 4:** Mean and 97.5th percentile hepatocarcinoma incidence (HCCi) values of the different age groups for 2019–2022, calculated with a probabilistic method.

Age groups	HCCi mean	HCCi P97.5
Toddlers	0.00022	0.00037
Children	0.00014	0.00023
Adolescents	0.00007	0.00012
Adults	0.00006	0.00010
Elderly	0.00005	0.00008

## Discussion

### Validation of results with deterministic method

As a validation step, the above-presented calculations were also performed with a semi-deterministic method which is described in the publication of Kerekes et al. (2021). Due to the nature of the method, the results were slightly higher, especially for the younger age groups at the high percentiles (Tables 5, 6), but confirmed that the probabilistic calculations were correct.

**Table 5:** Mean and 97.5th percentile (P97.5) margin of exposure (MoE) values of the different age groups for 2019–2022 calculated with a semi-deterministic method.

Age groups	MoE mean	MoE P97.5
Toddlers	34,219	11,854
Children	47,981	17,096
Adolescents	105,320	43,032
Adults	168,352	57,931
Elderly	215,673	74,611

**Table 6:** Mean and 97.5th percentile (P97.5) hazard index (HI) values of the different age groups for 2019–2022 calculated with a semi-deterministic method.

Age groups	HI mean	HI P97.5
Toddlers	0.58	1.69
Children	0.42	1.17
Adolescents	0.19	0.46
Adults	0.12	0.35
Elderly	0.09	0.27

### Main findings of the study

The project demonstrated, that in general, the Hungarian consumers, except for the large consumer group of toddlers, were not at considerable health risk from AFM1 exposure in the last 4 years. However, if earlier monitoring results are included (2011–2022) in our study and the focus is extended, the outcomes (Figures 5, 6; Tables 7, 8) look more worrying. When in the growing season, extremely high aflatoxin B1 concentrations occurred in maize to be used as cattle feed (as it happened, e.g., in 2012) (Tóth et al., 2013), it results in high AFM1 occurrence in milk and milk products. This shifted the calculated 97.5th percentile HI calculations in a range that negatively affects the results for not only the group of toddlers but the group of children as well. Even with the MoE method, the 97.5th percentile value of toddlers reached the level, which is of concern. This example clearly demonstrates that aflatoxin values need to be closely monitored on the long run. More data are necessary regarding the occurrence of AFM1 in processed milk products. The consumption habits of the Hungarian infants also need to be evaluated in order to assess the exposure of this age group as well.

**Table 7:** Hazard index (HI) values, calculated from the median of the mean and 97.5th percentile (P97.5) exposure distributions of the different age groups for the period 2011–2022.

Age groups	HI mean	HI P97.5
Toddlers	0.78	1.68
Children	0.49	1.05
Adolescents	0.25	0.52
Adults	0.21	0.46
Elderly	0.18	0.74

**Table 8:** Margin of exposure (MoE) values, calculated from the median of the mean and 97.5th percentile (P97.5) exposure distributions of the different age groups for the period 2011–2022.

Age groups	MoE mean	MoE P97.5
Toddlers	25,510	11,873
Children	40,568	19,048
Adolescents	81,301	38,573
Adults	94,118	43,384
Elderly	113,960	27,009

### Exposure assessment and risk characterization results and approaches

The occurrence of AFM1 is a worldwide problem. In certain cases, it can reach extremely high contamination (Saha Turna and Wu, 2021), and, for some areas, the increase of the contamination can also be seen in the published data, especially in African (Zinedine et al., 2021) and Asian countries (Nejad et al., 2019; Xiong et al., 2021). It should be pointed out that the maximum limits are not health-based limits but reflect the maximum concentration of a contaminant that can be maintained under the growing conditions of the country. That explains the large difference among ML values. Moreover, a generally small percentage of marketed commodities contain AFM1 at or above the ML.

Regarding the exposure assessment studies, Sumantri et al. (2019) stated that in Indonesia, all consumer age groups are highly exposed to AFM1; however, the AFM1 levels of the consumed food products did not exceed the Indonesian regulatory limits. This is a great example for pointing out the fact that exposure of consumers may not only derive from high contamination levels but also from high consumption of foodstuffs contaminated with low aflatoxin concentration. In a study from Ghana, a risk assessment on raw cow milk samples revealed a public health concern with about 50% of the samples exceeding the regulatory limit (Kortei et al., 2022). An AFM1 study on milk in Iran (Nejad et al., 2019) shows a bad practice for exposure estimation as the authors applied the European maximum permitted limit as a tolerable level to calculate HI values from EDI, which is not an appropriate approach that serves consumer health as AFM1 is a carcinogenic compound for which maximum permitted level is not equivalent to the tolerable intake level. Despite that around 85% of the tested milk samples was contaminated with AFM1, the authors concluded that there was no consumer risk deriving from milk consumption which is misleading. For HI calculations for AFM1, the use of the approach presented by Kuiper-Goodman (1990) instead of maximum limits gives far more realistic results for hazard characterization. In a study from Greece (Malissiova et al., 2022), AFM1 exposure exceeded the safe levels for infants and toddlers even with the use of maximum limits for HI calculations. In our study, AFM1 was non-quantifiable in almost 80% of the milk samples, and even with this low proportion level of contamination, the results of the hazard characterization indicated that a part of the youngest age groups are at risk of AFM1 exposure. The risk of the youngest age groups was also the conclusion of the study of Roila et al. (2021) from the Central Italian region and the studies of Kerekes et al. (2016) and Serraino et al. (2019) also related to the Italian population. AFM1 risk of consumers of broader age groups is presented in other European studies (from Greece, Albania, and Serbia) as well (Udovicki et al., 2019; Djekic et al., 2020; Milićević et al., 2021; Topi et al., 2022) which underlines the emergence and the importance of the topic.

As the prevalence of hepatitis B is low in Hungary (and in Europe in general), the aflatoxin-induced increase in HCCi does not show high values either. This conclusion is in line with the results of Saha Turna et al. (2022). Although the numerical value of the estimated incidence of liver cancer proved to be very low, the relative values of the results in this case also show a higher risk for toddlers and children compared to other age groups.

### Future research directions

According to conclusions of studies focusing on European areas, AFM1 is generally of lesser concern in this region; however, for assessing consumer exposure, hazard characterization often underestimates the consumer risk as EDI values are often compared to maximum limits. These results must be handled with care as they do not reflect health risk of consumers properly. Unified, sophisticated methods for intake assessments (such as probabilistic methods) and risk characterization of carcinogens are of paramount importance. The results of our and many other publications show that there is a health concern of long-term consumption of milk and milk products regarding AFM1, especially in case of younger age groups. But besides milk products, many other foodstuffs such as cereals and nuts are likely to contain high amounts of aflatoxins and other mycotoxins which may potentiate the effects of each other. Judging the risk of aflatoxin intake only by AFM1 exposure assessment underestimates the true health risk as AFB1 contributes ten times more to consumer exposure than AFM1. The biochemistry and the metabolic pathways of multi-mycotoxin effects are yet to be studied just as the methodology for the exposure assessment of multi-mycotoxin contamination.

## Data availability statement

The data analyzed in this study are subject to the following licenses/restrictions: The dataset can only be used with the permission of the authors. Requests to access these datasets should be directed to ZF, farkas.zsuzsa@univet.hu.

## Author contributions

ÁA, IP, ÁJ, PS, and MS contributed to the conception and design of the study. AN, TP, FP, GM, and AL organized the data collection and provided data for the study. SC performed the distribution analysis and run the Monte Carlo simulations. KK performed the deterministic exposure assessment and summarized and plotted the results. ZF and KK wrote the first draft of the manuscript. ZF finalized the manuscript and prepared for publication. ZF and KK contributed equally to this work and share first authorship. All authors contributed to manuscript revision and read and approved the submitted version. IP, AN, and MS contributed with funding acquisition of the study.

## Funding

This work was supported by Project no. 2018-1.2.1-NKP-2018-00002 which been implemented with the support provided from the National Research, Development and Innovation Fund of Hungary, financed under the 2018-1.2.1-NKP funding scheme; and Project no. RRF-2.3.1-21-2022-00001 which has been implemented with the support provided by the Recovery and Resilience Facility (RRF), financed under the National Recovery Fund budget estimate, RRF-2.3.1-21 funding scheme.

## Conflict of interest

The authors declare that the research was conducted in the absence of any commercial or financial relationships that could be construed as a potential conflict of interest.

## Publisher’s note

All claims expressed in this article are solely those of the authors and do not necessarily represent those of their affiliated organizations, or those of the publisher, the editors and the reviewers. Any product that may be evaluated in this article, or claim that may be made by its manufacturer, is not guaranteed or endorsed by the publisher.

## Abbreviations

AFB1: aflatoxin B1
AFB2: aflatoxin B2
AFG1: aflatoxin G1
AFG2: aflatoxin G2
AFM1: aflatoxin M1
AIC: Akaike’s information criterion
BCT: Box-Cox t distribution
BIC: Bayesian information criterion
BMDL_10_: benchmark dose lower confidence limit for a 10% response
DE: University of Debrecen
EDI: estimated daily intake
EFSA: European Food Safety Authority
ELISA: enzyme-linked immunoassay
HCCi: hepatocellular carcinoma incidence
HI: hazard index
HPLC: high-performance liquid chromatography
KNIME: Konstanz information miner
LOD: limit of detection
LOQ: limit of quantification
MoE: margin of exposure
NÉBIH: National Food Chain Safety Office
OIM: observed individual mean.
